# The immune-modulatory dynamics of exosomes in preeclampsia

**DOI:** 10.1007/s00404-025-08013-7

**Published:** 2025-04-03

**Authors:** M. David, N. Maharaj

**Affiliations:** Department of Obstetrics and Gynaecology, School of Clinical Medicine, Faculty of Health Sciences, University of the Free State, Bloemfontein, South Africa

**Keywords:** Preeclampsia, Exosomes, Immunological mechanisms

## Abstract

This review delves into the role of exosomes in immune regulation within the context of preeclampsia (PE), a pregnancy condition marked by high blood pressure and widespread inflammation. PE hampers the invasion of trophoblasts and disrupts placental function, contributing to inflammation and maternal organ dysfunction. Exosomes are small extracellular vesicles that mediate cell-to-cell communication by transferring proteins, lipids, and nucleic acids. This review highlights their role in immune regulation during pregnancy, especially their altered behavior in PE. Normally, exosomes support communication between the mother and fetus, promoting immune tolerance. In PE, however, exosomal activity and content undergo significant changes, potentially intensifying the inflammatory state. Further investigation into the in vivo immune-modulatory actions of exosomes, especially those from preeclamptic placentas, may provide insights into the pathogenesis of PE and uncover novel therapeutic targets for treatment.

## Introduction

Preeclampsia (PE) is a multifactorial pregnancy disorder characterized by the new sudden onset of high blood pressure and systemic inflammation. It is a significant contributor to maternal and fetal complications and death worldwide [1]. Despite intensive research, the mechanisms behind PE remain poorly understood, making it a challenge for obstetric management. Recent advances in the study of extracellular vesicles, particularly exosomes, offer new perspectives on the molecular pathways involved in PE. Exosomes are nano-sized vesicles secreted by various cells that enable communication between cells by transferring bioactive substances such as proteins, lipids, and nucleic acids [2, 3].

During pregnancy, exosomes play an essential role in ensuring communication between the mother and fetus, modulating immune responses to maintain tolerance and protection [2]. However, in PE, exosome production and composition change drastically, contributing to inflammation and vascular dysfunction [4]. Exosomes have emerged as potential biomarkers for early PE detection and possible therapeutic targets.

This review aims to provide an overview of the current understanding of how exosomes contribute to PE by examining their formation, content, and function in both normal and preeclamptic pregnancies. In addition, it stresses the need for more research into the immunoregulatory role of exosomes in PE to clarify their involvement in the disease and explore their therapeutic potential.

## The origin and function of exosomes

Exosomes are extracellular vesicles (Evs) ranging from 40 to 160 nm in size, secreted by nearly all cell types. Their formation occurs in endosomal compartments called multivesicular bodies (MVBs), which result from the inward budding of the endosomal membrane [5]. Exosomes carry various molecules, such as lipids, proteins, DNA, mRNAs, and microRNAs, which contribute to their biogenesis and transport. These vesicles house different proteins, including membrane-associated and surface proteins, which are crucial for their function [6, 7].

**Fig. 1:**
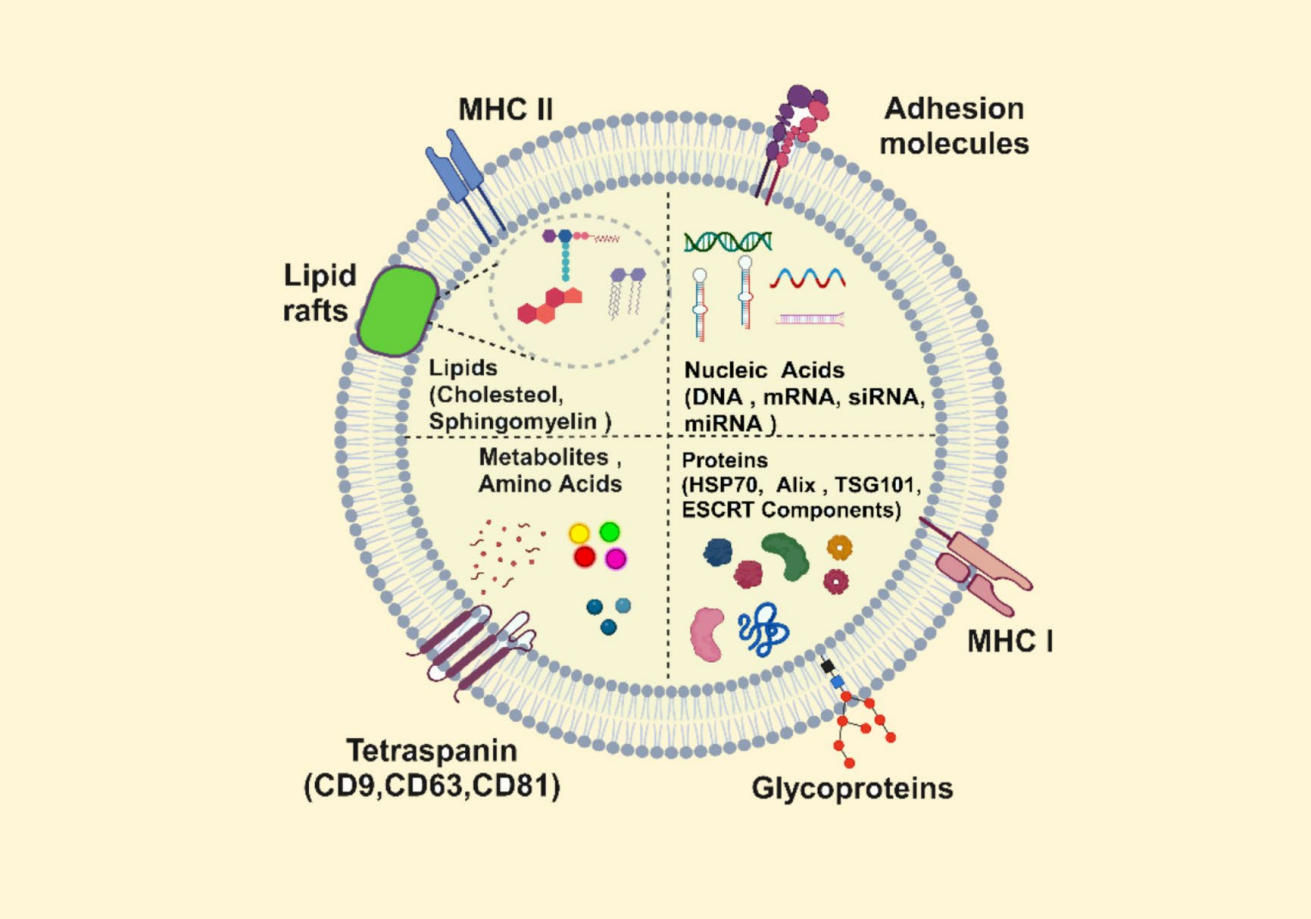
A schematic diagram of exosomes. They possess a double-membrane structure similar to that of the cell membrane. Exosomes encapsulate a variety of bioactive molecules, including proteins, lipids, RNAs, and DNAs. They also contain specific proteins such as tetraspanins (e.g., CD9, CD63, CD81), heat shock proteins (HSP70, HSP90), integrins, and components of the ESCRT complex

The release of extracellular vesicles is mediated by several proteins [8]. Among these are tetraspanins and ESCRT (endosomal sorting complex required for transport) proteins (Fig. 1), essential for the formation of exosomes [9]. Tetraspanins, like CD9, CD63, and CD81, are often used as biomarkers for exosomes [10].

MVBs can be formed via two pathways: one dependent on ESCRT proteins and another that works independently. When MVBs fuse with the cell membrane, their contents are released as exosomes into the extracellular space [11]. The ESCRT complex is made up of about 30 distinct proteins that are essential for endocytosis. The endosomal sorting complex necessary for transport is a protein complex that creates networks to facilitate protein sorting and the formation of MVB vesicles. These vesicles are considered intraluminal vesicles when placed within MVBs; when the contents of MVBs are discharged into the extracellular environment through cell membrane exocytosis, they are denoted as exosomes [8, 9, 12].

## Function of exosomes

Exosome functions vary depending on the cell or tissue origin and include immunologic response, antigen presentation, programmed cell death, angiogenesis, inflammation, coagulation, and morphogen transporters in forming polarity during development and differentiation [13]. Exosomes are highly specialized in transporting proteins, lipids, and nucleic acids to other cells, influencing their behavior and mediating genetic material transfer through protein interactions on their surface [14]. Exosomes are distributed throughout various bodily fluids, making them potential biomarkers. In addition, they serve as delivery vehicles for therapeutic agents, capable of crossing biologic barriers to reach target cells [15].

## Exosome isolation and characterization methods

Exosomes can be isolated and characterized utilizing a variety of approaches, each having unique strengths and limitations in terms of yield, purity, and specificity. The three most widely utilized methods are ultracentrifugation, size-exclusion chromatography (SEC), and affinity-based approaches [16–18].

*1. Ultracentrifugation*: Ultracentrifugation is considered the gold standard for exosome isolations and is extensively used due to its capability of separating exosomes based on their size and density. The process involves sequential centrifugation steps at increasing speeds to pellet larger debris and cells, followed by ultracentrifugation to isolate exosomes.

*Advantages*: Allows for large sample volumes to be processed and provides relatively high yields of exosomes.

*Limitations*: The risk of contaminating exosomes with non-vesicular particles such as protein aggregates or apoptotic bodies. Furthermore, it requires specialized equipment and is time-consuming. It may also compromise the exosome integrity due to high g-forces applied over prolonged periods.

*2. Size-exclusion chromatography (SEC)*: SEC separates exosomes based on their size by passing the samples through a column packed with porous beads. Larger vesicles elute first, whilst the smaller molecules, such as proteins, remain in the column longer. SEC is increasingly used for isolating higher-purity exosomes, especially for functional studies.

*Advantages*: it preserves exosome integrity and functionality, allowing for downstream applications such as proteomics and RNA analysis. Achieves higher purity compared to ultracentrifugation by reducing contamination with proteins and other soluble factors.

*Limitations*: The yield may be lower compared to ultracentrifugation, especially for smaller sample volumes, and multiple rounds of SEC may be required to obtain sufficient exosome quantities.

*3. Affinity-based techniques*: Affinity-based methods involve the use of antibodies or ligands that bind specific markers on the surface of exosomes (e.g., tetraspanins such as CD63, CD81, and CD9). This method can selectively capture exosome subpopulations with distinct molecular markers.

*Advantages*: High specificity for isolating exosome subpopulations, allowing for detailed characterization of vesicle origin and cargo and is suitable for low-volume samples.

*Limitations*: Risk of bias towards specific exosome subtypes based on marker selection, potentially excluding other relevant EV populations. Higher cost and complexity compared to traditional isolation methods.

## Immunomodulatory aspects of exosomes

**Fig. 2:**
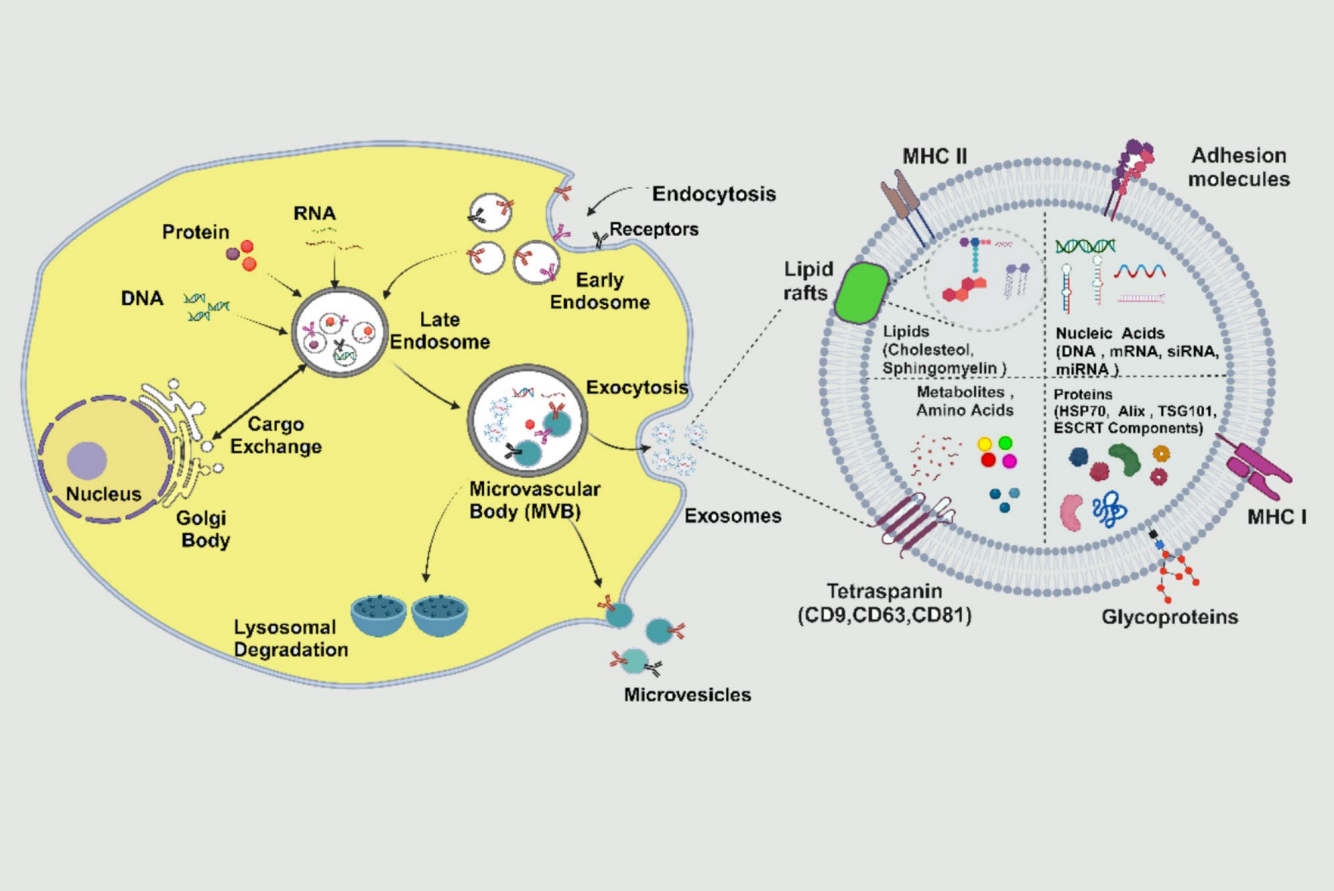
Schematic diagram of the immunomodulatory function of Exosomes. Exosomes play a key immunomodulatory role by transporting bioactive molecules, such as proteins, lipids, and RNA, which can influence immune cell communication and responses

Exosomes play an important role in immune regulation by presenting and transferring antigenic peptides. They carry peptides from antigen-presenting cells (APCs) that activate T lymphocytes [19]. These APCs carry peptide MHC-II and costimulatory signals. Exosomes containing IFN-α and IFN-γ, tumor necrosis factor a (TNF-α), and IL from macrophages induced dendritic cell maturation, CD4 + and CD8 + T cell activation, and macrophage IL expression regulation [4]. APCs carrying MHC-II and costimulatory signals on exosomes can stimulate T-cell responses. Exosome cargo, including DNA and miRNA, regulates immune responses and gene expression, which influences immune cell development (Fig. 2) [20].

Exosomes are essential in facilitating intercellular communication, influencing processes like inflammation, coagulation, and cell death [21]. Exosomes derived from dendritic cells, B lymphocytes and tumor cells can modulate immune memory by presenting antigens on MHC molecules, thus activating T cells. They also play a key role in delivering functional MHC-peptide complexes, impacting immune responses [22]. Exosomes are critical in transporting and delivering functional MHC-peptide complexes, which modify antigen-specific CD8 + and CD4 + responses [23].

Inflammation is a necessary process for maintaining homeostasis in biologic systems. Exosomes appear to play an essential part in inflammation processes by transporting cargo molecules such as miRNA and proteins that act on both proximal and distant target tissues.

Exosomes serve as carriers for miRNAs, which are endogenous non-coding RNA molecules that facilitate intercellular communication and control protein production while avoiding destruction in the adverse extracellular environment. Exosomes preserve miRNAs from degradation, allowing them to be transported to recipient cells to alter target gene expression and biologic functions [24]. Extracellular miRNAs such as miR-146a and miR-21 play a variety of roles in apoptosis, proliferation, cell migration, inflammation and immune regulation [24, 25].

In immunological modulation, miR-146a and miR-21 are especially significant. MiR-146a has been linked to the control of inflammatory reactions. By targeting important molecules from the Toll-like receptor (TLR) signaling cascade, such as TNF receptor-associated factor 6 (TRAF6) and IL-1 receptor-associated kinase 1 (IRAK1), it reduces the inflammatory response. MiR-146a delivered exosomally can aid in reducing excessive inflammation, which is essential for preserving immunologic homeostasis [26, 27].

Similarly, miR-21 has been demonstrated to modulate immunological responses, particularly in cancer and chronic inflammation [21]. miR-21 has been shown to impact processes such as apoptosis and proliferation, and exosomal transfer can influence immune cell activity, including macrophages. In immune cells, miR-21 increases the M2 macrophage phenotype, which is linked to anti-inflammatory responses and tissue healing [25, 26]. Combined, these miRNAs carried by exosomes help regulate immune responses, making them possible therapeutic targets in inflammatory disorders such as PE.

Exosomes have been reported to have a dual function in inflammasome activation, namely impacting the NLRP3 inflammasome, which is critical for controlling inflammatory responses. The NLRP3 inflammasome is a cytoplasmic protein complex that responds to pathogen- or damage-associated molecular patterns (PAMPs or DAMPs) [28]. When activated, the NLRP3 inflammasome cleaves pro-caspase-1 into caspase-1, which stimulates pro-inflammatory cytokines IL-1β and IL-18 and causes pyroptosis, an inflammatory form of cell death. Exosomes can modulate and carry signals that activate or inhibit the NLRP3 inflammasome. Exosomes from lipopolysaccharide (LPS) or nigericin-treated cells can activate inflammasomes in receiving cells, leading to increased production of IL-1β [28, 29].

Bioactive compounds found in exosomes, such as lipids, amino acids, and metabolites, have a significant effect on immune functioning by controlling both inflammatory and anti-inflammatory pathways. It has been observed that phosphatidylserine (PS), a lipid frequently present in the outer leaflet of exosomal membranes, is essential for immunologic regulation [30]. By encouraging the removal of apoptotic cells and suppressing pro-inflammatory reactions, PS on the surface of exosomes aids in immune evasion and establishes an anti-inflammatory environment. In addition, exosomal metabolites are important for immunological control. In this regard, it has been reported that exosomes produced from mesenchymal stem cells in hypoxic environments include immunomodulatory compounds like lactate, which can affect macrophage polarization and T-cell activation [29, 31]. Furthermore, amino acids transported by exosomes have the ability to alter immune cell activity and cellular metabolism, which helps to regulate inflammatory responses [28].

## Preeclampsia (PE)

Preeclampsia is a pregnancy-related condition characterized by new-onset hypertension (BP ≥ 140 mmHg systolic or ≥ 90 mmHg diastolic) after 20 weeks of gestation [1]. It is associated with maternal organ dysfunction, proteinuria, and systemic inflammation, which can lead to severe complications like liver and kidney dysfunction, as well as “HELLP” syndrome [32, 33]. Globally, the prevalence of PE is estimated to be 2–4%, with 46,000 maternal deaths and 500,000 fetal and newborn deaths annually [1].

Preeclampsia can be divided into two subtypes: early and late-onset PE [34]. Clinical indications of early-onset PE (EOPE) arise before 33 gestational weeks and are related to deficient placentation, whereas late-onset PE (LOPE) appears after 34 weeks of gestation. Late-onset PE accounts for over 80% of preeclamptic cases [35]. The EOPE is linked to a variety of severe consequences, including face edema, abdominal wall edema, disseminated intravascular coagulation, liver failure, renal failure, central nervous system abnormalities, and an increased risk of organ system dysfunction. LOPE is linked to an imbalance between placental senescence and maternal susceptibility to cardiovascular and metabolic problems [36].

## Pathogenesis of PE

**Fig. 3:**
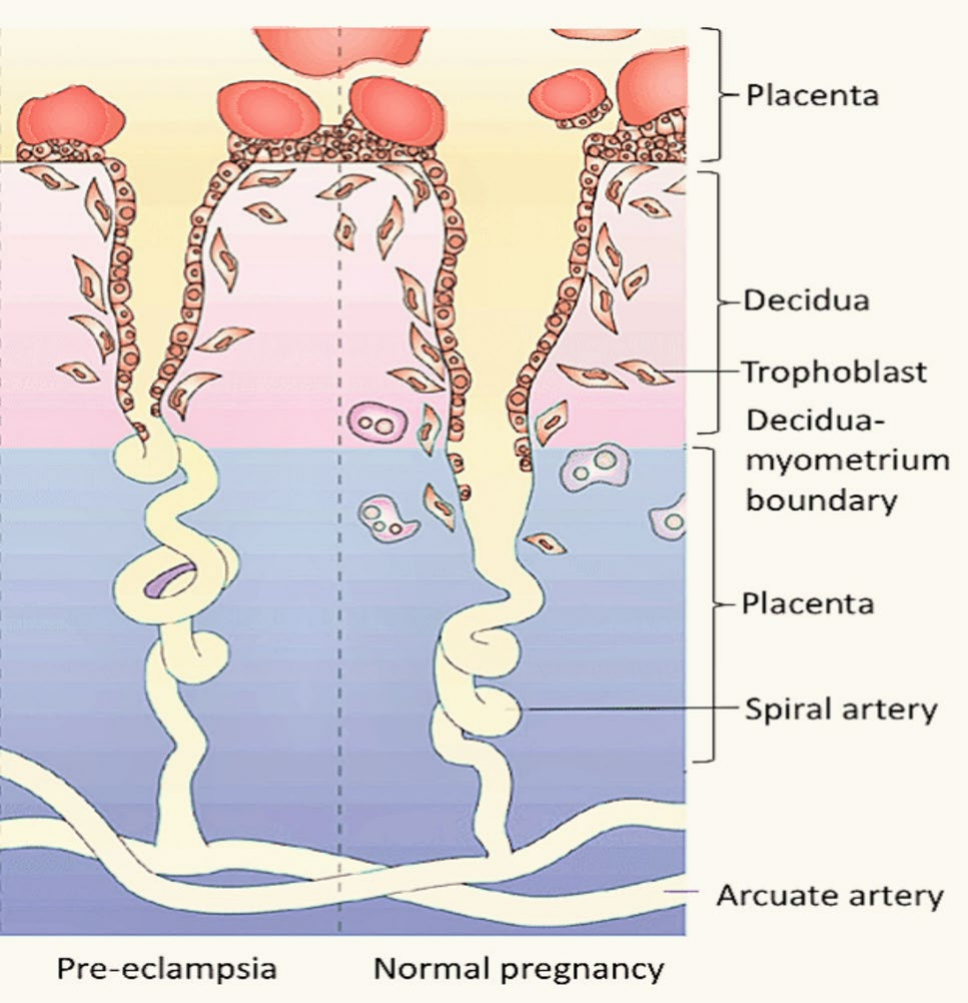
Schematic overview of the pathogenesis of preeclampsia. Inadequate migration of extravillous trophoblast (EVT) cells results in shallow trophoblast invasion, therefore reducing spiral artery remodeling and overall lack of nutrients to the fetus. Adapted from Bergman, 2021 [41]

Although the exact causes of PE remain unclear, it is thought to involve abnormal migration of extravillous trophoblast (EVT) cells, leading to inadequate remodeling of the spiral arteries [37]. This is associated with a reduction in the diameter of blood vessels, resulting in an insufficient blood supply and nutrition requirement for the development of the fetus [38]. Furthermore, this causes hypoxia in the placenta, which triggers the release of anti-angiogenic factors such as soluble endoglin, soluble fms-like (sFlt-1) and inflammatory mediators into the maternal bloodstream [39, 40]. These factors contribute to the hyper-inflammatory state observed in PE Fig. 3.

## Role of T lymphocytes and macrophage dynamics during pregnancy and PE

T lymphocytes, particularly regulatory T (Treg) and T helper 17 (Th17) cells are essential for maintaining immunologic balance during pregnancy. Tregs are critical in developing maternal–fetal tolerance as they inhibit hyperactive immune responses that can harm the growing fetus. TGF-β and IL-10, two anti-inflammatory cytokines produced by these cells, support the immunosuppressive milieu at the maternal–fetal interface [42]. In addition, Tregs prevent effector T cell activation, which helps to regulate immunological responses during pregnancy [43]. Immune tolerance is disrupted in preeclampsia due to a substantial decline in Treg function and quantity. It is proposed that this decrease has a role in placental inflammation, which results in inadequate trophoblast invasion, aberrant placentation, and the clinical signs of PE that follow.

Th17 cells, on the other hand, are pro-inflammatory and frequently linked to chronic inflammation and autoimmune disorders. Inflammatory cytokines, including TNF-α, IL-6, and IL-17, are produced by Th17 cells and results in inflammation and tissue damage [44].

The quantity and activity of Th17 cells are elevated in preeclampsia. Immune dysregulation in PE is characterized by this imbalance, which involves decreased Treg function and increased Th17 activity. Endothelial dysfunction, hypertension, and systemic inflammation are all exacerbated by the increased inflammatory cytokines caused by the heightened Th17 response [45].

During normal pregnancy, the innate and adaptive immune systems adapt to prevent fetal rejection and protect against invading pathogens while safeguarding the mother. This process involves a transition from a T-helper 1 (TH1) to a T-helper 2 (TH2) type immune response, which favors an immuno-tolerant milieu [46]. However, in PE, this shift does not occur, compromising maternal tolerance to the fetus and resulting in increased neutrophil and monocyte activity. Notably, monocytes produce large amounts of pro-inflammatory chemokines and cytokines. Furthermore, CD8 + , CD4 + , and dendritic cells exacerbate the pro-inflammatory response [47].

During normal pregnancy, M2 macrophages predominate. These cells are engaged in anti-inflammatory responses, tissue remodeling, and angiogenesis, all of which are necessary for normal placental development and maintenance. M2 macrophages release anti-inflammatory cytokines, including IL-10 and TGF-β, which promote vascularization and trophoblast invasion at the maternal–fetal interface [44]. In preeclampsia, M1 macrophages shift toward a pro-inflammatory phenotype. M1 macrophages release inflammatory cytokines such as IL-1β, IL-6, and TNF-α, leading to placental inflammation and endothelial dysfunction [48]. This shift is associated with poor placental development and increased oxidative stress, which are the hallmark features of PE.

## Role of exosomes during Pregnancy and PE

The placenta is a key regulator of maternal adaptation to pregnancy, releasing various types of extracellular vesicles, including exosomes, to communicate with maternal tissues. [49]. During pregnancy, the placenta, particularly the syncytiotrophoblasts, produces several sizes of extracellular vesicles, including macrovesicles (Syncytial Nuclear Aggregates; 20–100 µm), microvesicles (0.2–1 µm), and nanovesicles (30–150 nm), which contain exosomes [3, 50].

Syncytiotrophoblasts, cells within the placenta, constitute a significant source of exosomes during pregnancy. These vesicles contain bioactive molecules, such as proteins and nucleic acids, influencing maternal physiology and fetal development. This process aids in adapting maternal physiology to support the rising needs of the developing fetus. Pregnancy is an excellent model for studying EVs because of its well-defined timeline, specific markers such as placental alkaline phosphatase (PLAP) that distinguish STB-derived extracellular vesicles from those of other cell origins, and the placenta’s accessibility as the source of these vesicles at the end of pregnancy [50, 51]. This emphasis on extracellular vesicles, like exosomes, is relevant to pregnancy problems like preeclampsia, which affects women worldwide and is a significant contributor to maternal mortality.

Preeclampsia (PE) is a pregnancy condition distinguished by hypertension and endothelial dysfunction, which is frequently related to the increased release of placental-derived exosomes (PdE) into the maternal circulation. PdEs can stimulate vascular endothelial cells, leukocytes, and platelets, resulting in endothelial dysfunction and systemic inflammation [51]. Low-dose aspirin (LDA) has been demonstrated to affect the release and composition of PdEs, impacting PE pathogenesis. According to studies, LDA medication may diminish the release of pro-inflammatory PdEs, potentially decreasing endothelial dysfunction and inflammation in PE patients [52].

Furthermore, it has been noted that in patients at cardiovascular risk, LDA temporarily lowers the levels of circulating extracellular vesicles (EVs), particularly platelet-derived EVs. This decrease in EVs is associated with cyclooxygenase-1 (COX-1) activity suppression, underscoring the effect of LDA on EV release and its potential significance in regulating inflammation and endothelial function [53, 54].

Moreover, PP13 (placental protein 13) is a syncytiotrophoblast-specific protein that has a role in placental vascular development and immunologic regulation. The dysregulation of PP13 expression and release via exosomes has been linked to aberrant placentation and vascular pathology in PE [55]. PP13 levels are also being considered as an early biomarker for predicting the beginning of PE [51]. By incorporating this marker, we hope to highlight the significance of syncytiotrophoblast-derived exosomes in vascular dysfunction and aberrant placental implantation.

Extravillous trophoblasts (EVTs) are essential for the invasion of the maternal decidua and modification of the spiral arteries, which ensures adequate blood supply to the placenta. Recent research suggests that EVTs release exosomes containing immunomodulatory proteins [56]. Notably, HLA-G is a prominent hallmark of EVT-derived exosomes, which promote immunological tolerance at the maternal–fetal interface by interacting with maternal immune cells and preventing rejection of the semi-allogeneic fetus [55]. These exosomes could contribute to the dysregulated immunologic responses seen in preeclampsia (PE).

**Fig. 4:**
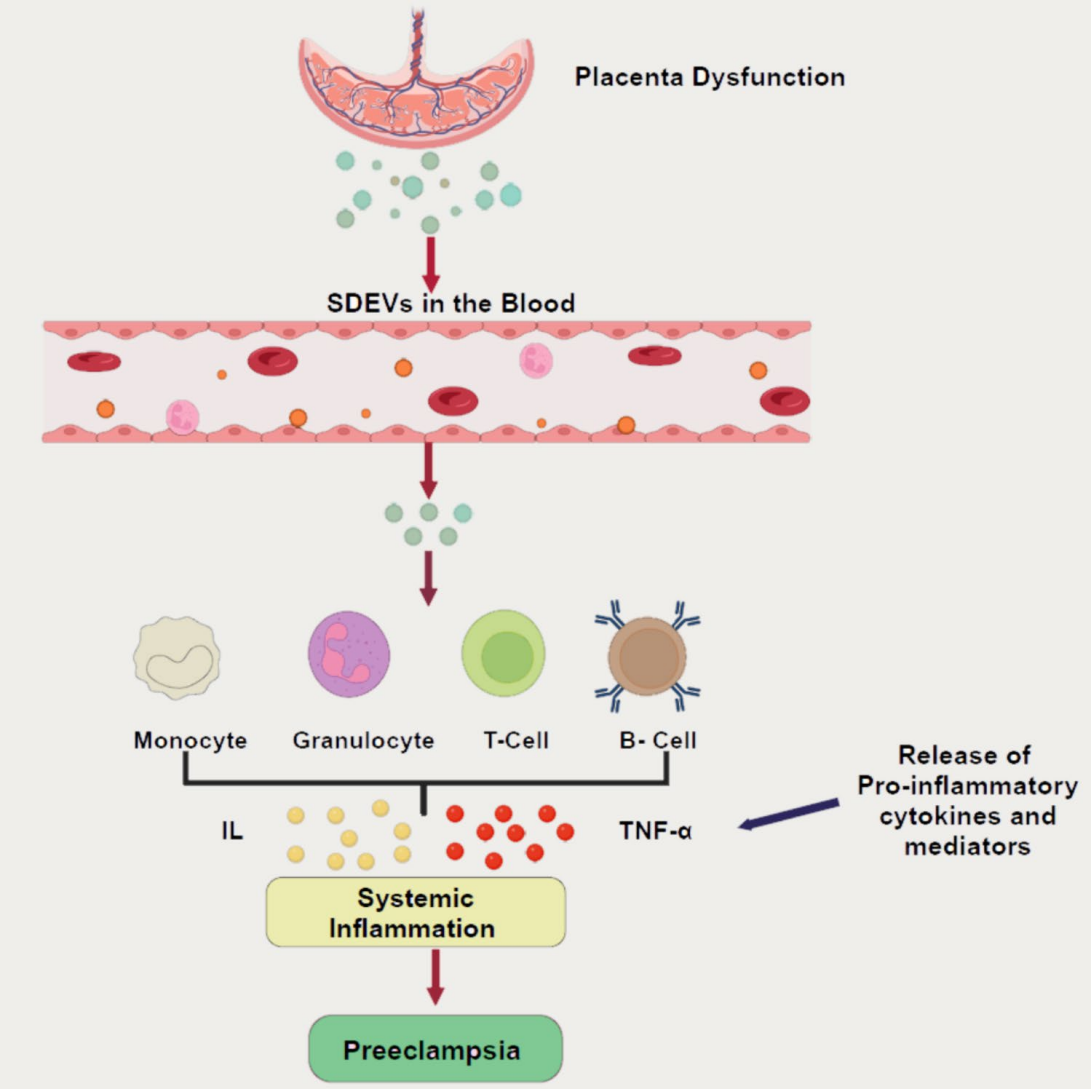
Schematic illustration of exosomes in the pathophysiology of PE. Exosomes are released from various placental cell types, including syncytiotrophoblast-derived extracellular vesicles (SDEVs), syncytiotrophoblasts, extravillous trophoblasts (EVTs), cytotrophoblasts, and maternal decidual cells, all of which contribute to exosome release into the maternal circulation and may play roles in preeclampsia (PE) pathophysiology. Numerous inflammatory mediators are elevated in preeclampsia, such as interleukins (e.g., IL-6, IL-1β), tumor necrosis factor-alpha (TNF-α), reactive oxygen species (ROS), and pro-inflammatory lipid mediators like prostaglandins and leukotrienes, which exacerbate inflammation and promote endothelial dysfunction. These exosomes collectively influence PE development

Maternal exosomes, notably those produced from immune cells such as leukocytes and vascular endothelial cells, have been linked to the systemic inflammation and endothelial dysfunction that characterize PE. Exosomes generated from leukocytes include pro-inflammatory cytokines, which can enhance the maternal immune response and promote chronic inflammation (Fig. 4). Similarly, endothelial cell-derived exosomes carry adhesion molecules and procoagulant factors that may aggravate endothelial damage, leading to hypertension and vascular issues related to PE.

Vargas et al. [35] conducted a study investigating the importance of Syncytin proteins integrated into placental exosomes. The study specifically focused on their function in cell uptake and examined differences in abundance in serum exosomes from patients diagnosed with preeclampsia. Their findings suggested that syncytin proteins are essential for promoting the cellular uptake of placental exosomes [57]. Trophoblasts release exosomes during pregnancy. The profile of growth factors, DNA fragments, mRNAs, miRNAs, various proteins, and phospholipids found in trophoblast-derived exosomes are found [34, 35].

PLAP functions as a distinct indicator for exosomes of syncytiotrophoblasts. First-trimester observations of PLAP-containing exosomes reveal a marked rise in their quantity as the pregnancy continues. These placental exosomes deliver a wide variety of biomolecules to their intended organs, including lipids, proteins, and miRNA [58]. The microenvironment of the placenta has a crucial role in controlling the content, production, and release of placental exosomes. For example, research has demonstrated that trophoblast cells produce more exosomes when exposed to hypoxic environments or increased glucose levels [3].

Maternal exosomes, particularly those produced by leukocytes and endothelial cells, are primarily responsible for maternal systemic inflammation, immunological activation, and endothelial dysfunction [59]. These pathways are essential for the development of hypertension, proteinuria, and multiorgan involvement in PE. In addition, Fetoplacental-derived exosomes, such as those from syncytiotrophoblasts and EVTs, play a larger role in placental development and immunologic modulation along the maternal–fetal interface. Their dysregulation can result in decreased placental perfusion, hypoxia, and the release of substances that worsen maternal PE symptoms [59, 60].

Notably, obesity and T2DM are major risk factors for PE and causes persistent low-grade inflammation, which affects exosome composition and function. Adipose tissue-derived exosomes (ADEs) from obese persons include pro-inflammatory adipokines like TNF-α, IL-6, and MIF [51]. These ADEs can activate macrophages, causing the release of more pro-inflammatory cytokines and contributing to insulin resistance. For example, exosomes from adipocytes have been demonstrated to stimulate M1 polarization of macrophages via the sonic hedgehog (Shh) signalling pathway, increasing inflammation and insulin resistance in adipose tissue [53]. Furthermore, in T2DM, pancreatic β-cell-derived exosomes transporting microRNA-29 (miR-29) can enhance the recruitment and activation of monocytes and macrophages, leading to systemic inflammation and affecting glucose homeostasis. This miR-29-mediated pathway highlights the importance of β-cell-derived exosomes in regulating immunological responses [52].

## Immunological role of exosomes during pregnancy and PE

In PE, exosome secretion has been reported to be substantially elevated than in normal pregnancy, whilst other investigations, however, showed a decreased number of exosomes in preeclamptic patients [38, 41, 44]; these studies used plasma samples to detect exosome concentrations. The inconsistency between these studies may be attributed to the methodology used. The investigations that discovered higher levels of exosomes in PE used an enzyme-linked immunosorbent test to assess exosomes [37]. This approach recognizes vesicles of all sizes. In PE, exosome levels are higher, and molecular load is slightly altered compared to during normal pregnancy [61]. Preeclamptic exosomes may disclose more molecules, including tissue factor, endoglin, and Flt-1. This could suggest that the exosome's functionality has been altered during PE. However, it is still unknown how the molecular load of the vesicles is coordinated and which factors determine the variations in the molecular load of normal and preeclamptic exosomes [61, 62].

As previously mentioned, exosomes provide several immunologic functions. Research suggests that exosomes have a role in pregnancy-related immune responses, as well as pregnancy-related disorders [63, 64]. In vitro and in vivo investigations have demonstrated that exosomes may play a role in the activation of the innate immune system during pregnancy, with monocytes able to bind and internalize exosomes [65].

Exosomes have been found in a variety of body fluids, including urine and saliva, and they have the potential to serve as non-invasive biomarkers for PE. Urinary exosomes can provide crucial information on both placental failure and renal involvement in the disease. These exosomes include proteins, miRNAs, and other biomolecules that represent the pathophysiology of PE [66]. Exosomes isolated from the urine of women with PE have been found to have higher quantities of angiogenic and anti-angiogenic substances such as sFlt-1 (soluble Fms-like tyrosine kinase-1) and PlGF (placental growth factor). These variables are important in the regulation of placental vasculature [67]. Higher levels of sFlt-1 in urine exosomes have been related to the angiogenesis imbalance that causes endothelial dysfunction and placental hypoxia, both hallmark characteristics of PE [63, 64].

Salivary exosomes have received less attention but are becoming recognized as potential biomarkers for pregnancy-related hypertension diseases, such as PE [68]. The systemic physiologic changes that occur in pregnant women are believed to be reflected in salivary exosomes [68, 69]. Significant changes in the miRNA content of salivary exosomes were found in women with PE, according to a recent study by Javadi et al. [70]. Salivary exosomes from PE patients showed a substantial increase in miRNAs associated with inflammation and oxidative stress, including miR-210. Since miR-210 is known to alter cellular responses to hypoxia, its presence in salivary exosomes may be a sign of oxidative damage and placental hypoxia, two key aspects of PE pathophysiology [65].

**Fig. 5:**
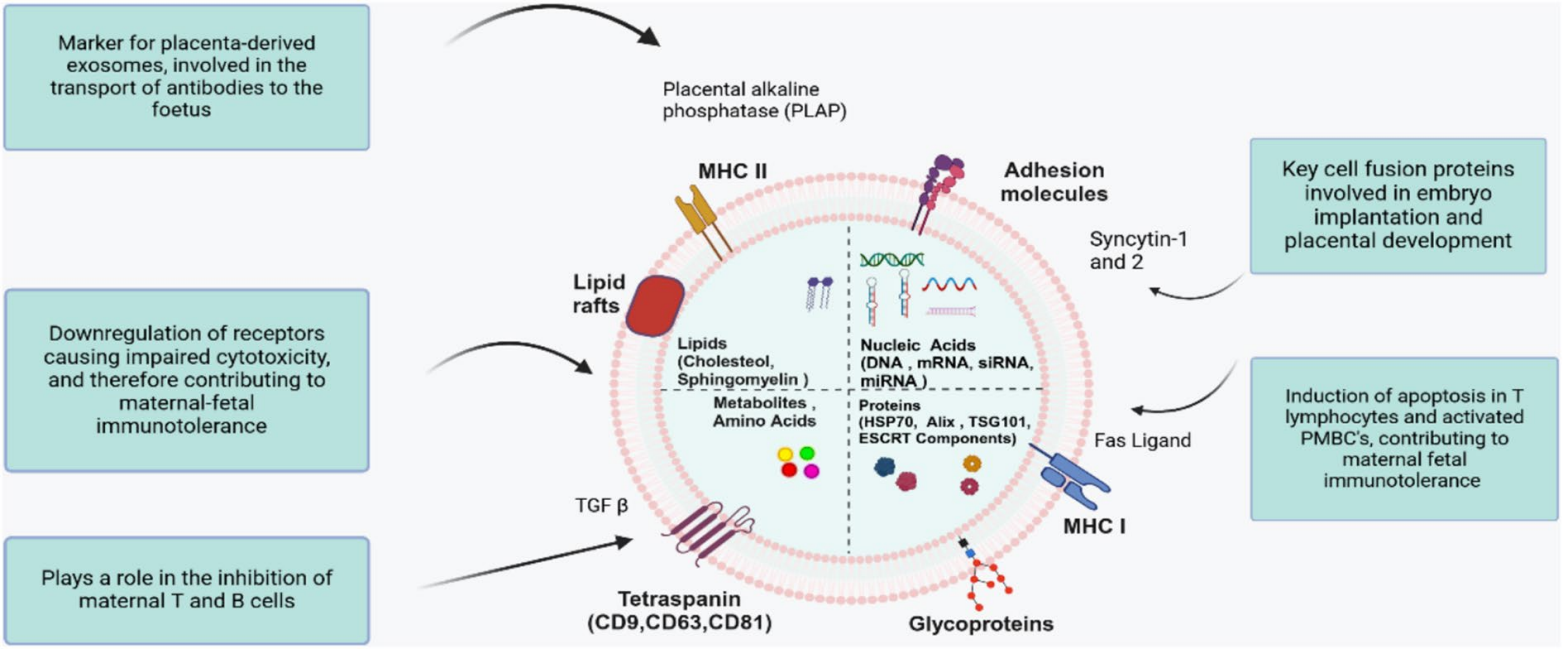
Schematic diagram of key pregnancy-associated exosome molecular cargo and its function. Exosomes, marked by placental alkaline phosphatase (PLAP), facilitate antibody transfer and carry molecules like MHC I, Fas ligand, and Syncytin proteins. These molecules induce apoptosis in maternal immune cells and aid in placental development. Lipid rafts and tetraspanins further modulate immune responses, including inhibiting maternal T and B cells. Glycoproteins and nucleic acids influence cell signalling and gene expression [66, 67]

After attaching to the exosomes, monocytes produce cytokines, including TGF β (Fig. 5), TNF-α and IL-1-β. Furthermore, neutrophils appear to be triggered by STB EV in vitro, as it has been demonstrated that neutrophils treated with exosomes create greater quantities of superoxide [36, 39]. These factors contribute to excess inflammation, creating a hyper-inflammatory state observed in PE [71].

Exosomes have been shown to bind to T and B cells and enhance phosphorylation of signal transducer and activator of transcription 3 in T cells in vitro, potentially impacting the adaptive immune response during pregnancy (Fig. 5) [72]. Exosomes also elevate interferon γ production in T cells. Furthermore, exosomes produced by phytohaemagglutinin can reduce T-cell proliferation. In addition, exosomes were discovered to reduce the allogeneic reactivity of T cells in mixed lymphocyte responses [8]. Exosomes derived from perfused normal placentae have been shown to have a regulatory function of activating activated Treg cells and memory T cells in vitro [73].

Exosomes in early-onset PE contain high quantities of anti-angiogenic factors and inflammatory cytokines such as sFlt-1, TNF-α and IL-6, and procoagulant molecules. High amounts of sFlt-1 in exosomes are thought to act via the VEGF signaling pathway, affecting normal angiogenesis by blocking VEGF binding to its receptor [74]. This inhibits the formation of placental blood vessels, resulting in placental hypoxia and aggravating the production of exosomes, which promote systemic inflammation and endothelial dysfunction. In contrast, exosomes from patients with late-onset PE typically contain lower quantities of anti-angiogenic and pro-inflammatory cytokines [72]. Instead, they are more likely to possess metabolic stress markers, such as miRNAs associated with insulin resistance and adipokines [75]. These exosomal profiles indicate that in late-onset PE, maternal metabolic health plays a larger role, with less severe placental impairment.

However, limited research has been conducted on the immunological impact of exosomes from preeclamptic placentae. Exosomes from preeclamptic placenta explant cultures are shown to increase pro-inflammatory cytokine release in peripheral blood mononuclear cells, including IL1-β, IL-6, IL-17, macrophage inhibitory protein-1-α and -β, and TNF-α, compared to normal placenta explants [8, 76]. Moreover, exosomes from preeclamptic placental explants stimulated the response of peripheral blood mononuclear cells to lipopolysaccharide, compared to exosomes from normal placental explants, which inhibited the response [64]. This suggests that exosomes from preeclamptic placentae may play a role in the increased inflammatory response seen in PE.

## Conclusion and further recommendations

Exosomes are a powerful communication pathway between the placenta and the mother body. Exosomes enter the maternal circulation in early pregnancy and increase throughout the pregnancy. Exosomes from healthy pregnant women have been shown in vitro to stimulate a variety of inflammatory cells, including monocytes and granulocytes, suggesting that they may play a role in establishing the physiologic general inflammatory state associated with pregnancy. Exosome numbers rise during PE, and their molecular load varies in comparison to a normal pregnancy. Indeed, exosomes from preeclamptic pregnancies may contribute to the hyper-inflammatory state that occurs following PE. While additional research is needed to completely understand their immunologic function in PE, exosomes show potential as non-invasive biomarkers for disease identification and surveillance. Their incorporation into standard prenatal screenings could result in earlier diagnosis and intervention. Furthermore, exosome-based therapeutics could be developed to control inflammation and minimize endothelial dysfunction, providing novel therapeutic options for improving maternal outcomes in PE. However, only a few studies have looked at the immunologic involvement of preeclamptic placenta exosomes. As a result, we need to better understand the role of exosomes in both normal and preeclamptic pregnancies. Future studies should focus on the in vivo immunomodulatory function of exosomes and their molecular differences, particularly from preeclamptic placentae; as well as investigate the clinical applicability of exosomal biomarkers and treatments in controlling PE.

## Data Availability

No datasets were generated or analysed during the current study.
